# GM-CSF Exhibits Anti-Inflammatory Activity on Endothelial Cells Derived from Chronic Venous Disease Patients

**DOI:** 10.1155/2013/561689

**Published:** 2013-11-13

**Authors:** Veronica Tisato, Paola Secchiero, Erika Rimondi, Sergio Gianesini, Erica Menegatti, Fabio Casciano, Paolo Zamboni, Giorgio Zauli

**Affiliations:** ^1^Department of Morphology, Surgery and Experimental Medicine and LTTA Centre, University of Ferrara, Via Fossato di Mortara 70, 44100 Ferrara, Italy; ^2^Department of Life Sciences, University of Trieste, Via Manzoni 16, 34137 Trieste, Italy; ^3^Vascular Disease Center, University of Ferrara, Via Aldo Moro 8, 44124 Ferrara, Italy; ^4^Institute for Maternal and Child Health, IRCCS Burlo Garofolo, Via dell'Istria 65/01, 34137 Trieste, Italy

## Abstract

Twenty patients affected by chronic venous disease (CVD) in tertiary venous network and/or saphenous vein were analyzed before surgical ablation by echo-color-doppler for the hemodynamic parameters reflux time (RT) and resistance index (RI), a negative and a positive prognostic factor, respectively. RT and RI were next correlated with relevant *in vitro* parameters of venous endothelial cells (VEC) obtained from surgical specimens, such as cell migration in response to serum gradient, proliferation index, intercellular adhesion molecule (ICAM)-1 and vascular cell adhesion molecule (VCAM)-1 expression, as well as cytokines release. Of interest, ICAM-1 expression in patient-derived VEC cultures correlated positively with RT and negatively with RI. Moreover, RT showed a positive correlation with the baseline osteoprotegerin (OPG) expression by VEC and an inverse correlation with VEC proliferation index. On the other hand, RI correlated positively with TNF-related apoptosis inducing ligand (TRAIL) expression. Among the cytokines released by VEC, GM-CSF showed a positive correlation with VEC proliferation and TRAIL expression and a negative correlation with OPG, ICAM-1 and VCAM-1 expression. Since *in vitro* recombinant GM-CSF induced VEC proliferation and counteracted the induction of ICAM-1, VCAM-1 and OPG upon exposure to TNF-**α**, our data suggest an anti-inflammatory activity of GM-CSF on venous endothelial cells.

## 1. Introduction

Venous endothelial cells (VEC) are normally exposed to hemodynamic forces, which modulate endothelial cells functions and vascular biology/pathobiology in health and disease [1, 2]. In the venous system, disturbed flow resulting from reflux, outflow obstruction, and/or stasis characterizing chronic venous disease (CVD) leads to venous inflammation and thrombosis [3, 4]. In the clinical context, echo-color Doppler (ECD) analysis allows the evaluation of clinically relevant hemodynamic parameters such as in particular the reflux time (RT) and the resistance index (RI). While higher reflux time implies more severe disease, and therefore RT is a negative prognostic factor for CVD, the RI is known to be related to vascular resistance and is therefore considered a positive prognostic factor in chronic venous insufficiency of the lower limbs [5, 6].

Understanding the effects of disturbed flow on VEC can provide mechanistic insights into the role of complex flow patterns in the pathogenesis of vascular diseases and can help to elucidate the phenotypic and functional differences between quiescent (nonthrombogenic) and activated (thrombogenic) VEC.

Although several reports have addressed the role of disturbed flow on endothelial cell activation using *in vitro* models of shear stress [1, 2], no data are available so far about the biological alterations of endothelial cells induced by altered blood flows *in vivo* characterizing CVD. In this context, some authors have proposed that cultured endothelial cells represent a suitable model for studying the inflammatory/thrombogenic properties of endothelium of various locations [7, 8]. In this respect, we have recently developed a protocol for the isolation and *ex vivo* characterization of VEC from large veins of patients at various stages of CVD [9, 10]. This experimental setting has allowed the evaluation of several *in vitro* biological features of pathological VEC, such as, in particular, cell migration, proliferation, expression of the proinflammatory markers, such as osteoprotegerin (OPG), ICAM-1, and VCAM-1 adhesion molecules [9], and release of cytokines under normal culture conditions or in response to proinflammatory stimulation upon exposure to TNF-*α* [10]. In this context, it is interesting to note that besides its role in osteoclastogenesis, OPG also acts as neutralizing receptor for TNF-related apoptosis inducing ligand (TRAIL), a TNF family member which has been previously shown to display protective activity on endothelial cells [11–14]. Moreover, it has been shown that, OPG also exhibits pro-inflammatory activity on endothelial cells [15], independently of its ability to neutralize TRAIL.

On these bases, the aim of the present study was to investigate potential correlations between the clinically relevant hemodynamic parameters of the patients from which the VEC were isolated (RT and RI) and *in vitro* biological features of the patient-derived VEC in order to identify key factors likely involved in modulating the *in vivo* biological features of VEC. For this purpose, we have also analysed the *in vitro* release of a panel of cytokines by VEC, which might play a role in promoting angiogenesis and tissue regeneration, including GM-CSF [16–19].

## 2. Material and Methods

### 2.1. Clinical and Demographic Characteristics of Patients

Twenty patients affected by primary CVD with superficial venous reflux (C2-3EpAsPr following the CEAP classification) undergoing varicose veins surgery were assessed by echo-color Doppler (ECD) for the following hemodynamic parameters: peak systolic velocity (PSV) and end diastolic velocity (EDV), which allowed the calculation of the resistance index (RI) and reflux time (RT). The echo-color Doppler has been made according to international guidelines. Main clinical and demographic characteristics of patients are summarized in Table 1. The venous segments, which were subsequently surgically ablated, were used for VEC isolation, by adopting the previously described protocol [9]. The procedures followed were in accordance with the Declaration of Helsinki, approved by the institutional review board (University Hospital of Ferrara) and all participant subjects gave written informed consent.

### 2.2. Cell Cultures and Treatments

After isolation from surgical segments, highly purified VEC cultures obtained from the CVD patients were characterized for morphological and phenotypic properties, as previously described [9]. VEC were grown on EGM2 medium (Lonza, Walkersville, MD) with 2% FBS and full supplements (EGM2 Bullet kit, Lonza) in 5 *μ*g/cm^2^ fibronectin precoated tissue culture plates (BD, Becton Dickinson, San Josè, CA) and used within passages 3 to 6, as previously reported [15]. 

For *in vitro* endothelial cell treatment, recombinant TNF-*α* (R&D Systems, Minneapolis, MN) and GM-CSF (PeproTech, NJ, USA) were used at the optimal concentration of 5 ng/mL and 20 ng/mL, respectively, as determined in preliminary dose-response experiments. In selected experiments recombinant GM-CSF was added to the cells 1 hour before exposure to TNF-*α*. After the different treatments, cell viability was monitored by light microscopic analysis of the cell monolayers, followed by quantitative examination by means of trypan blue dye exclusion, and apoptosis was evaluated by annexin V/propidium iodide (PI) staining followed by flow cytometry analysis, as previously reported [20–22]. 

### 2.3. Cell Proliferation Assay

Cell proliferation was performed as previously described [9] using the DP version of the xCELLigence real time cell analyzer RTCA (Roche Diagnostics, Mannheim, Germany), which records changes in impedance (reported as a cell index-CI) over a prolonged time course in a noninvasive system. Briefly, the background impedance of RTCA DP E-Plates 16 was performed using the standard protocol provided in the software with 100 *μ*L of EGM-2. Five thousand endothelial cells were seeded in quadruplicate in fibronectin pre-coated wells with 100 *μ*L of complete (2% FBS and cytokines/growth factors) EGM2 and left to equilibrate at room temperature for 30 minutes. Cells were allowed to grow for 22 hours before adding GM-CSF to the cultures at the indicated concentrations. The CI of the proliferating cells was recorded and expressed as mean of CI normalized to the CI recorded at the time of GM-CSF cell treatment compared to untreated cells.

### 2.4. Real-Time Reverse Transcription-PCR Analysis

Real-time reverse transcription-(RT)-PCR analysis was adopted to quantitatively compare the expression levels of ICAM-1, VCAM-1, TRAIL, and OPG in VEC cultures. Total RNA was extracted from cells by using RNeasy Plus mini kit (Qiagen, Hilden, Germany) according to the supplier's instructions. The quality of the total RNA preparation was verified by agarose gel and, when necessary, further purification was performed with the RNeasy cleanup system (Qiagen) to remove chromatin DNA. Total RNA was transcribed into cDNA, using the QuantiTect Reverse Transcription kit (Qiagen). Analysis of ICAM-1, VCAM-1, TRAIL, and OPG gene expression was carried out using the SYBR Green real-time PCR detection method with the SABiosciences RT2 Real-Time Gene expression assays that include specific validated primer sets and PCR master mixes (SABiosciences, Frederick, MD), as previously described [23]. All samples were run in triplicate by using the real time thermal analyzer rotor-gene 6000 (Corbett, Cambridge, UK). Expression values were normalized to the housekeeping gene POLR2A amplified in the same sample.

### 2.5. Multiplex Immunoassay

VEC culture supernatant samples were frozen and thawed only once before performing the analysis of the cytokines/chemokines by using the human cytokine 27-Bio-Plex assay (BioRad Laboratories, Milan, Italy), a bead-based multiplex immunoassay. Samples were processed in duplicate following the supplier's instructions and read on a Bio-Plex 200 instrument equipped with the software Bioplex Manager, using a five-parameter not-linear regression formula to compute sample concentrations from the standard curves.

### 2.6. Assessment of Cell Surface Inflammatory Markers

The surface expression of the inflammatory markers ICAM-1 and VCAM-1 was analyzed by flow cytometry. In brief, cells were detached with trypsin-EDTA, washed and 5 × 10^5^ cells were resuspended in 200 *μ*L of PBS containing 1% BSA (Sigma-Aldrich, St Louis, MO) and incubated 30 minutes at 4°C with the following monoclonal antibodies (mAb): FITC-conjugated anti-ICAM-1 (R&D, Clone BBIG-I1) or FITC-conjugated anti-VCAM-1 (R&D, Clone BBIG-V3). Nonspecific fluorescence was assessed by incubation with isotype-matched conjugated mAb [20, 24].

### 2.7. Enzyme-Linked Immunosorbent Assay (ELISA) for OPG Measurement

Endothelial culture supernatants were analyzed for OPG release by using ELISA kits (Alexis Biochemicals, Lausen, Switzerland) according to the manufacturer's instructions and as previously described [11]. Measurements were done in duplicate and the results were read at an optical density of 450 nm using an Anthos 2010 ELISA reader (Anthos Labtec Instruments Ges.m.b.H). Sensitivity of the OPG assay was 2.8 pg/mL, the intra- and interassay coefficients of variation (CV) were 9% and <10%, respectively.

### 2.8. Statistical Analysis

Descriptive statistics were calculated. For each set of experiments, values were reported as means ± SD and box plots were used to show the median, minimum, and maximum values and 25th to 75th percentiles. The results were evaluated by using Student's *t*-test and the Mann-Whitney rank-sum tests, when appropriate. Spearman's correlation coefficient was calculated to identify data correlation. Statistical significance was defined as *P* < 0.05. All statistical analyses were performed with SPSS Statistic 20 software (SPSS Inc., Chicago, IL).

## 3. Results

### 3.1. Correlation between *In Vivo* Relevant Hemodynamic Parameters and *In Vitro* Biological Features of Patient-Derived VEC

In a previous study, we have identified and characterized several biological parameters of VEC obtained from surgical specimens of CVD patients undergoing surgery for conservative and hemodynamic treatment of venous insufficiency in ambulatory care (CHIVA) [9]. Since we had the availability of the hemodynamic measurements performed into the venous segments that were surgically ablated and from which we derived pure vein endothelial cultures (Table 1), in this study we have performed a set of correlation analyses between key *in vitro* biological characteristics of VEC (spontaneous cell migration, proliferation index and baseline expression levels of ICAM-1, VCAM-1, OPG, and TRAIL) and the two major hemodynamic parameters resistance index (RI) and reflux time (RT) (Table 2). As reported in Table 2 and shown in Supplementary Figure 1 in the Supplementary Material available online at http://dx.doi.org/10.1155/2013/561689, we found that the expression of ICAM-1 in VEC correlated negatively with RI (*P* = 0.002) and positively with RT (*P* = 0.007) measured in the same CVD patients from which the VEC were isolates. Moreover, we found a negative correlation between *in vitro* VEC proliferation and RT (*P* = 0.027), while the expression of OPG correlated positively with RT values (*P* = 0.013). Moreover, there was a significant (*P* = 0.048) positive correlation between RI expression and TRAIL expression.

### 3.2. Release of GM-CSF by Patient-Derived VEC Correlates with Cell Proliferation and Expression of Inflammatory Markers

**Figure 1:**
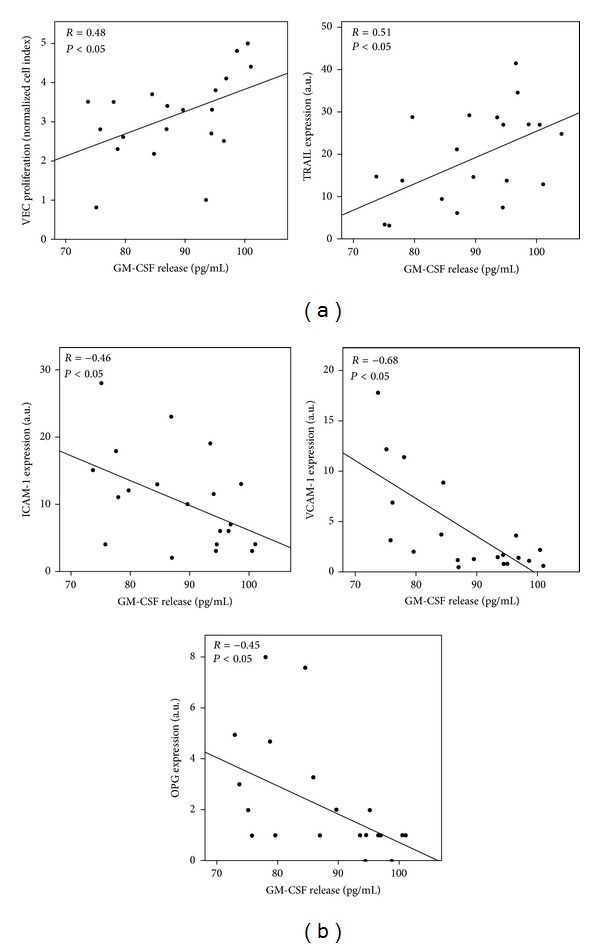
Correlation analyses between spontaneous release of GM-CSF and *in vitro* biological features of VEC cultures. In (a), positive correlation between baseline release of GM-CSF and *in vitro* cell proliferation index and between baseline release of GM-CSF expression and TRAIL expression in VEC cultures. In (b), negative correlation between baseline release of GM-CSF and levels of expression of ICAM-1, VCAM-1, and OPG in VEC cultures. Correlation coefficients (*R*), calculated by Spearman's analysis, are reported for each correlation.

We next analysed the levels of a panel of 27 cytokines/growth factors spontaneously released by CVD patient derived cultured VEC in order to identify soluble factors, which might have a role in modulating the observed biological parameters. Of interest, among the detectable cytokines spontaneously released by VEC, only the levels of GM-CSF showed a statistically significant positive correlation with the spontaneous endothelial cell proliferation (*R* = 0.48; *P* = 0.02) (Figure 1(a)), in line with previous findings highlighting the role of GM-CSF in promoting angiogenesis and tissue regeneration [16, 19]. Moreover a significant positive correlation was found with the expression levels of TRAIL (*R* = 0.51; *P* = 0.04) (Figure 1(a)). In the same fashion, the spontaneous release of GM-CSF by pathological VEC negatively correlates with the expression levels of both the expression of ICAM-1 (*R* = −0.46; *P* = 0.04) and VCAM-1 (*R* = −0.68; *P* = 0.02), as well as with of the pro-inflammatory cytokine OPG (*R* = −0.45; *P* = 0.02) (Figure 1(b)). Therefore, these analyses indicated that GM-CSF showed a positive correlation with those biological parameters (endothelial cell proliferation and TRAIL expression), which were related to a better prognosis (RI values) and an inverse correlation with those parameters (ICAM-1, VCAM-1, and OPG expression), which were related to a worse prognosis (RT values) as indicated in Table 2.

### 3.3. Recombinant GM-CSF Increases the *In Vitro* Cell Proliferation and Attenuates the TNF-*α* Induced Expression of the Pro-Inflammatory Markers

**Figure 2:**
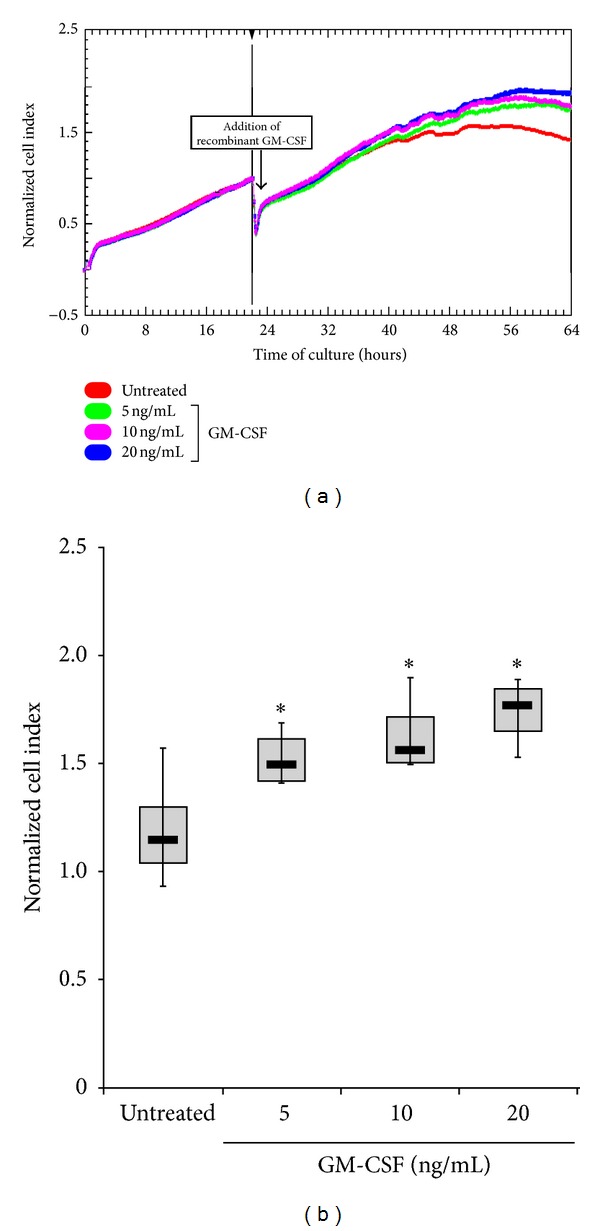
GM-CSF treatment induces *in vitro* VEC proliferation. Dynamic monitoring of endothelial cells proliferation using the xCELLigence system. VEC were seeded at a density of 5.000 cells/well per well in quadruplicate in fibronectin-coated 16 wells E-plates and left adhere for 22 h when recombinant GM-CSF was added to the cultures at the indicated concentrations. In (a), cell proliferation was recorded and expressed as cell index (CI) after normalization to the CI recorded at the time of GM-CSF addition (arrow). A representative panel of GM-CSF-treated and untreated cell proliferation expressed as normalized CI is shown. In (b), the dose-dependent effect of GM-CSF in inducing VEC proliferation is shown as normalized CI of result from 4 independent VEC cultures. Horizontal bars are median, upper, and lower edges of box are 75th and 25th percentiles; lines extending from box are 10th and 90th percentiles. *P < 0.05 compared to untreated VEC.

The correlations illustrated above suggested, but did not prove, that GM-CSF might have an anti-inflammatory role in pathological VEC. Therefore, we first investigated whether recombinant GM-CSF was able to affect the *in vitro* proliferation rate of CVD-derived pathological VEC. In fact, although it has already been reported that GM-CSF might have proangiogenic activity [25, 26], no information are available so far about the effect of this molecule on pathological VEC isolated from CVD patients. The proliferative response of VEC to increasing concentration of recombinant GM-CSF (up to 20 ng/mL) was investigated using the real-time and label-free monitoring of cell proliferation based on electrical impedance real-time cell analysis (RTCA). As shown in Figures 2(a) and 2(b), VEC proliferation/survival was increased in a dose-dependent manner by the addition in culture medium of GM-CSF, with the maximum effect at the dose of 20 ng/mL (Figure 2(b)).

**Figure 3:**
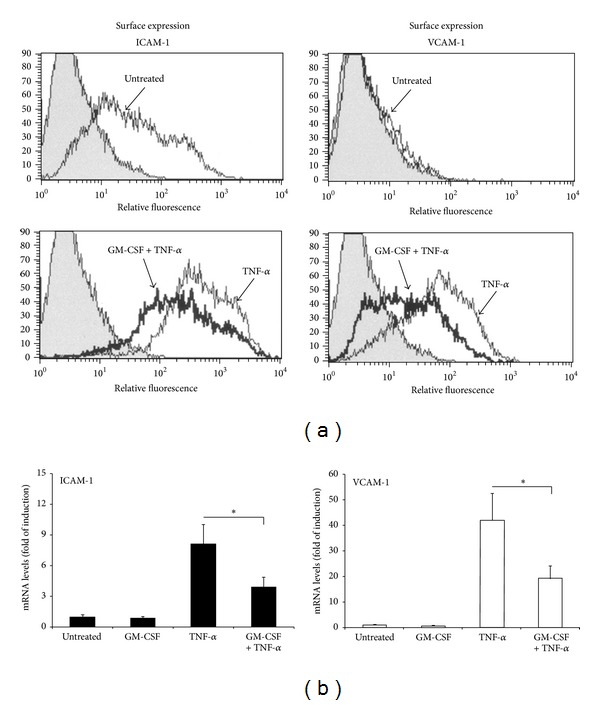
GM-CSF treatment downmodulates the TNF-*α* induced expression of ICAM-1 and VCAM-1. VEC were left untreated or pretreated for 1 hour with GM-CSF (20 ng/mL) before addition of TNF-*α* (5 ng/mL). ICAM-1 expression and VCAM-1 expression were analyzed after 24 hours of TNF-*α* stimulation. In (a), surface expression of ICAM-1 and VCAM-1 was analyzed by flow-cytometry. White histograms represent cells stained with monoclonal antibodies specific for the indicated antigens and grey histograms represent background mean fluorescence intensity obtained by staining the same cells with isotype-matched control antibodies. A representative panel for ICAM-1 expression and VCAM-1 expression in cultures, treated as indicated, is shown. In (b), the expression levels of ICAM-1 and VCAM-1 in VEC cultures treated as indicated were determined by quantitative RT-PCR. Results from amplifications, done in duplicate, are expressed as fold of untreated cells (set to 1), after normalization for the housekeeping gene. *P < 0.05.

In light of the fact that pathological VEC are exposed *in vivo* to a pro-inflammatory *milieu*, in additional experiments CVD patient-derived VEC cultures were exposed *in vitro* to recombinant TNF-*α* in order to mimic the *in vivo* inflammatory microenvironment. In this setting, the baseline levels of surface ICAM-1 expression analysed by flow-cytometry were unaffected by recombinant GM-CSF treatment, while the levels of surface VCAM-1 expression were at the limit of detection. On the other hand, upon exposure to TNF-*α* surface expression of both ICAM-1 and VCAM-1 was markedly induced (Figure 3(a)). Of interest, in the presence of recombinant GM-CSF, the TNF-*α* induction of both ICAM-1 and VCAM-1 was significantly downmodulated (*P* < 0.05), (Figure 3(a)). Similar results were observed at transcriptional level, by analysing the ICAM-1 and VCAM-1 mRNA upon treatment with GM-CSF of TNF-*α*-stimulated VEC cultures (Figure 3(b)). 

**Figure 4:**
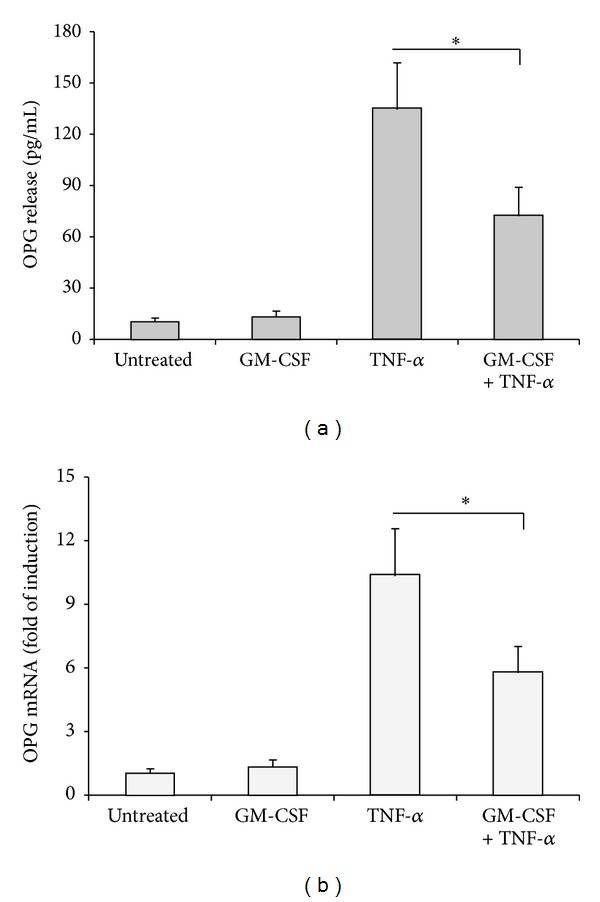
GM-CSF treatment downmodulates the TNF-*α* induced expression of the proinflammatory marker OPG. VEC were left untreated or pretreated for 1 hour with GM-CSF (20 ng/mL) before addition of TNF-*α* (5 ng/mL). OPG expression was analyzed after 24 hours of TNF-*α* stimulation. In (a), the release of OPG in supernatants of VEC cultures treated as indicated was analyzed by ELISA. Results are expressed as means + SD of result from 4 independent VEC cultures. *P < 0.05. In (b), the expression levels of OPG in VEC cultures treated as indicated were determined by quantitative RT-PCR. Results from amplifications, done in duplicate, are expressed as fold of untreated cells (set to 1), after normalization for the housekeeping gene. *P < 0.05.

Finally, in the last group of experiments, we have analysed whether GM-CSF was able to inhibit the release of the pro-inflammatory mediator OPG. As shown in Figure 4, the analysis of both OPG protein released in culture supernatants (Figure 4(a)), as well as of OPG mRNA levels (Figure 4(b)) after 24 hours of TNF-*α* stimulation showed a significant (*P* < 0.05) inhibition in the presence of GM-CSF (20 ng/mL) (Figures 4(a) and 4(b)).

## 4. Discussion

In light of the fact that large vein endothelium plays important roles in clinical diseases, such as CVD and thrombosis [27–31], the availability of a proper *in vitro* model able to resume the local and pathological-related changes by expressing and releasing specific cytokines, chemokines, and soluble mediators involved in the pathophysiology of these diseases might have a strategic role. We have previously demonstrated that pathological VEC isolated from CVD patients exhibit a peculiar pro-inflammatory phenotype, defined by specific *in vitro* biological characteristics [9]. Moreover, our subsequent findings in this model suggested the involvement of hemodynamic forces in the generation of the pro-inflammatory feature underlying, in particular, the contribution of soluble mediators, such as PDGF-BB [10]. 

In the attempt of a better definition of the link between relevant *in vivo* hemodynamic parameters and *in vitro* characteristics of pathological endothelial cells, in the present study we have demonstrated that the hemodynamic parameters RT and RI calculated into the venous segments from which the VEC were isolated, correlate with specific *in vitro* biological VEC features. In particular, we demonstrate that (i) RT values positively correlated with the VEC expression of ICAM-1 and of OPG and negatively correlated with the VEC proliferation index; (ii) RI values positively correlated with the VEC expression of TRAIL and negatively correlated with the expression of ICAM-1; (iii) the baseline release levels of GM-CSF by patient-derived VEC positively correlated with VEC proliferation and expression level of TRAIL and inversely correlated with the expression levels of ICAM-1, VCAM-1, and OPG. The direct effects of GM-CSF on endothelial cells were then investigated by using our *in vitro* model of pathological VEC. In particular, we have then demonstrated, that recombinant GM-CSF is able to promote VEC proliferation/survival and, in a pro-inflammatory milieu mediated by TNF-*α*, it can transcriptionally down-modulate the expression of the inflammatory markers ICAM-1, VCAM-1 which are known to be involved in the recruitment of leukocytes, platelets, and erythrocytes to the vein wall [32, 33]. Moreover, the demonstration that GM-CSF can significantly counteract the release of OPG, suggests that GM-CSF is able to interfere with the role of OPG in the vascular physiopathology, with significant clinical consequences since high circulating OPG levels are associated with endothelial dysfunction in several pathological conditions and represents a risk factor for cardiovascular disease progression [11, 34]. In this respect, we have previously demonstrated that the spontaneous release of OPG by pathological VEC is the consequence of higher baseline levels of NF-kB and thus reflects an inflammatory *in vivo* status of the endothelium of CVD patients [9]. 

Although originally identified as a hematopoietic growth factor, GM-CSF is produced by a wide range of cell types, including endothelial cells. In the context of our study, it is noteworthy that a previous report performed in the standard *in vitro* models of endothelium based on human umbilical vein endothelial cells (HUVEC), reported that the production of GM-CSF is regulated by hemodynamic forces [35]. Moreover it has been established that GM-CSF is able to induce and support endothelial cell proliferation, and tissue regeneration phenomena that may lead to endothelial capillary formation [16–19, 36]. However, the specific vascular sites from which endothelial cells belong to might have a crucial role on the cellular phenotype and biological behaviour of the cells. Therefore it is noteworthy that GM-CSF, endogenously produced by endothelial cells, can display anti-inflammatory activity on pathologic VEC. 

Although we are aware that a limitation of this exploratory analysis is the relatively small sample size examined, due to the complexity in obtaining pure VEC cultures from CVD patients which does not allow large scale studies [9], our results suggested a significant link between *in vivo* haemodynamic forces and *in vitro* pathological endothelial cell biology in the context of CVD and underlie the potential role of GM-CSF in modulating the pro-inflammatory response of CVD patient-derived VEC. On the basis of our observations, it remains to be established whether the use of recombinant GM-CSF, which is widely used in human therapy mainly in the oncology [37] and sepsis [38] fields, would represent a therapeutic tool for promoting clinical remission of CVD in alternative to surgical ablation.

## Supplementary Material

The following main *in vitro* biological features of VEC: cell migration, proliferation index and baseline expression levels of ICAM-1, VCAM-1, OPG and TRAIL, were analyzed in correlation with the key hemodynamic parameters resistance index (RI) and reflux time (RT). The analysis of the correlations showed that ICAM-1 expression in VEC cultures was negatively correlated with RI (p=0.002) and positively correlated with RT (p=0.007) (Supplementary Figure 1). The spontaneous *in vitro* VEC proliferation was negatively correlated with RT (p=0.027), while the expression of OPG positively correlated with RT values (p=0.013). Moreover, TRAIL expression levels showed a significant (p=0.048) positive correlation with RI (Supplementary Figure 1).

## Figures and Tables

**Table 1:** Main demographic and clinical data of the cohort of patients and phenotypic characteristics of the purified VEC.

Variable	Cohort (*n* = 20)*
Patient age (years)	57.8 ± 9.7
Men/women	5/15
Body mass index (kg/m^2^)	26.05 ± 4.11
Age at CVD onset (years)	36.15 ± 9.79
Relevant medical history: yes/no	
Family history of CVD	17/2
DM2	2/18
Cardiac disease	1/19
Hypercholesterolemia	4/16
Hypertension	9/11
Current smoker	5/15
CEAP classification: C2/C3	8/12
Varicose veins surgery: CHIVA 1/CHIVA 2	13/7
Hemodynamic parameters	
PSV (cm/s)	29 ± 13
EDV (cm/s)	8 ± 6
RI	1.31 ± 0.19
RT (s)	2.66 ± 0.46
VEC phenotypic properties:	
CD31^+^/CD105^+^	93–98%
CD146^+^/CD144^+^	90–98%
CD45^−^/CD14^−^	0–0.9%
Surface ICAM-1 (MFI)	64.12 ± 61.93
Surface VCAM-1 (MFI)	ND

*Values are expressed as mean ± SD unless otherwise indicated.

CVD: chronic venous disease; DM2: diabetes mellitus type 2; CEAP: clinical signs, etiology, anatomic distribution, and pathophysiology; CHIVA: cure hémodynamique de l'insuffisance veineuse en ambulatoire; PSV: peak systolic velocity; EDV: end diastolic velocity; RI: resistance index; RT: reflux time; MFI: mean fluorescence intensity; ND: not detectable.

**Table 2:** *In vitro* VEC biological features and correlation with *in vivo* hemodynamic parameters.

VEC biological features	Hemodynamic parameters	
	Resistance index (RI)	Reflux time (RT)
	*R* (*P* value)*	*R* (*P* value)*
Cell proliferation index	n.s.	−0.6 (0.027)
Cell migration index	n.s.	n.s.
Expression levels of ICAM-1	−0.4 (0.002)	0.4 (0.007)
Expression levels of VCAM-1	n.s.	n.s.
Expression levels of OPG	n.s.	0.5 (0.013)
Expression levels of TRAIL	0.4 (0.048)	n.s.

*Correlation coefficients were determined by Spearman's analysis; n.s.: not statistically significant.

## References

[1] Chiu JJ, Chien S (2011). Effects of disturbed flow on vascular endothelium: pathophysiological basis and clinical perspectives. *Physiological Reviews*.

[2] Li YS, Haga JH, Chien S (2005). Molecular basis of the effects of shear stress on vascular endothelial cells. *Journal of Biomechanics*.

[3] Chiu JJ, Usami S, Chien S (2009). Vascular endothelial responses to altered shear stress: pathologic implications for atherosclerosis. *Annals of Medicine*.

[4] Raffetto JD (2009). Dermal pathology, cellular biology, and inflammation in chronic venous disease. *Thrombosis Research*.

[5] Reček C (2006). Conception of the venous hemodynamics in the lower extremity. *Angiology*.

[6] Zamboni P, Franceschi C (2009). *Principles of Venous Hemodynamics*.

[7] Lang I, Pabst MA, Hiden U (2003). Heterogeneity of microvascular endothelial cells isolated from human term placenta and macrovascular umbilical vein endothelial cells. *European Journal of Cell Biology*.

[8] Eriksson EE, Karlof E, Landmark K, Rotzius P, Hedin U, Xie X (2005). Powerful inflammatory properties of large vein endothelium in vivo. *Arteriosclerosis, Thrombosis, and Vascular Biology*.

[9] Tisato V, Zauli G, Voltan R (2012). Endothelial cells obtained from patients affected by chronic venous disease exhibit a pro-inflammatory phenotype. *PLoS ONE*.

[10] Tisato V, Zamboni P, Menegatti E (2013). Endothelial PDGF-BB produced ex vivo correlates with relevant hemodynamic parameters in patients affected by chronic venous disease. *Cytokine*.

[11] Secchiero P, Corallini F, Beltrami AP (2010). An imbalanced OPG/TRAIL ratio is associated to severe acute myocardial infarction. *Atherosclerosis*.

[12] Zauli G, Melloni E, Capitani S, Secchiero P (2009). Role of full-length osteoprotegerin in tumor cell biology. *Cellular and Molecular Life Sciences*.

[13] Secchiero P, Zauli G (2008). Tumor necrosis factor-related apoptosis-inducing ligand and the regulation of hematopoiesis. *Current Opinion in Hematology*.

[14] Secchiero P, Gonelli A, Celeghini C (2001). Activation of the nitric oxide synthase pathway represents a key component of tumor necrosis factor-related apoptosis-inducing ligand-mediated cytotoxicity on hematologic malignancies. *Blood*.

[15] Zauli G, Corallini F, Bossi F (2007). Osteoprotegerin increases leukocyte adhesion to endothelial cells both in vitro and in vivo. *Blood*.

[16] Wong VW, Crawford JD (2013). Vasculogenic cytokines in wound healing. *BioMed Research International*.

[17] Grochot-Przeczek A, Dulak J, Jozkowicz A (2013). Therapeutic angiogenesis for revascularization in peripheral artery disease. *Gene*.

[18] Jin DK, Shido K, Kopp HG (2006). Cytokine-mediated deployment of SDF-1 induces revascularization through recruitment of CXCR4^+^ hemangiocytes. *Nature Medicine*.

[19] Grote K, Schuett H, Salguero G (2010). Toll-like receptor 2/6 stimulation promotes angiogenesis via GM-CSF as a potential strategy for immune defense and tissue regeneration. *Blood*.

[20] Re MC, Zauli G, Gibellini D (1993). Uninfected haematopoietic progenitor (CD34+) cells purified from the bone marrow of AIDS patients are committed to apoptotic cell death in culture. *AIDS*.

[21] Zauli G, La Placa M, Vignoli M (1995). An autocrine loop of HIV type-1 Tat protein responsible for the improved survival/proliferation capacity of permanently tat-transfected cells and required for optimal HIV-1 LTR transactivating activity. *Journal of Acquired Immune Deficiency Syndromes and Human Retrovirology*.

[22] Vitale M, Zamai L, Falcieri E (1997). IMP dehydrogenase inhibitor, tiazofurin, induces apoptosis in K562 human erythroleukemia cells. *Cytometry B*.

[23] Secchiero P, Melloni E, di Iasio MG (2009). Nutlin-3 up-regulates the expression of Notch1 in both myeloid and lymphoid leukemic cells, as part of a negative feedback antiapoptotic mechanism. *Blood*.

[24] Zauli G, Bassini A, Vitale M (1997). Thrombopoietin enhances the *α*(IIb)*β*3-dependent adhesion of megakaryocytic cells to fibrinogen or fibronectin through PI 3 kinase. *Blood*.

[25] Bussolino F, Ziche M, Wang JM (1991). In vitro and in vivo activation of endothelial cells by colony-stimulating factors. *Journal of Clinical Investigation*.

[26] Ebner K, Bandion A, Binder BR, de Martin R, Schmid JA (2003). GMCSF activates NF-*κ*B via direct interaction of the GMCSF receptor with I*κ*B kinase *β*. *Blood*.

[27] Wakefield TW, Strieter RM, Prince MR, Downing LJ, Greenfield LJ (1997). Pathogenesis of venous thrombosis: a new insight. *Cardiovascular Surgery*.

[28] Eberhardt RT, Raffetto JD (2005). Chronic venous insufficiency. *Circulation*.

[29] Müller AM, Hermanns MI, Cronen C, Kirkpatrick CJ (2002). Comparative study of adhesion molecule expression in cultured human macro- and microvascular endothelial cells. *Experimental and Molecular Pathology*.

[30] Lang I, Hoffmann C, Olip H (2001). Differential mitogenic responses of human macrovascular and microvascular endothelial cells to cytokines underline their phenotypic heterogeneity. *Cell Proliferation*.

[31] Eriksson EE, Karlof E, Landmark K, Rotzius P, Hedin U, Xie X (2005). Powerful inflammatory properties of large vein endothelium in vivo. *Arteriosclerosis, Thrombosis, and Vascular Biology*.

[32] Lawson C, Wolf S (2009). ICAM-1 signaling in endothelial cells. *Pharmacological Reports*.

[33] Alcaide P, Maganto-Garcia E, Newton G (2012). Difference in Th1 and Th17 lymphocyte adhesion to endothelium. *Journal of Immunology*.

[34] Montagnana M, Lippi G, Danese E, Guidi GC (2013). The role of osteoprotegerin in cardiovascular disease. *Annals of Medicine*.

[35] Kosaki K, Ando J, Korenaga R, Kurokawa T, Kamiya A (1998). Fluid shear stress increases the production of granulocyte-macrophage colony-stimulating factor by endothelial cells via mRNA stabilization. *Circulation Research*.

[36] Krubasik D, Eisenach PA, Kunz-Schughart LA, Murphy G, English WR (2008). Granulocyte-macrophage colony stimulating factor induces endothelial capillary formation through induction of membrane-type 1 matrix metalloproteinase expression in vitro. *International Journal of Cancer*.

[37] Horneber M, Milazzo S, Liu JP, Marcel Z, Birkmann J (2009). Colony-stimulating factors for the prevention of chemotherapy induced febrile neutropenia in breast cancer patients. *Cochrane Database of Systematic Reviews*.

[38] Bo L, Wang F, Zhu J, Li J, Deng X (2011). Granulocyte-colony stimulating factor (G-CSF) and granulocyte-macrophage colony stimulating factor (GM-CSF) for sepsis: a meta-analysis. *Critical Care*.

