# Supplementary material for: GM-CSF Exhibits Anti-Inflammatory Activity on Endothelial Cells Derived from Chronic Venous Disease Patients
Source: Mediators Inflamm. 2013 Nov 13;2013:561689. doi: 10.1155/2013/561689 (PMC3845402; doi:10.1155/2013/561689)

## Supplementary Material

**Supplementary Figure 1. Correlation analyses between *in vitro* VEC biological features and *in vivo* hemodynamic parameters.** Negative correlation between *in vitro* cell proliferation index and reflux time (RT) and between ICAM-1 expression in VEC cultures and resistance index (RI). Positive correlation between the levels of expression of ICAM-1 in VEC cultures and RT, between TRAIL expression in VEC cultures and RI and between OPG expression in VEC cultures and RT. Correlation coefficients (R), calculated by Spearman's analysis, are reported for each correlation.

## Supplementary Figure 1

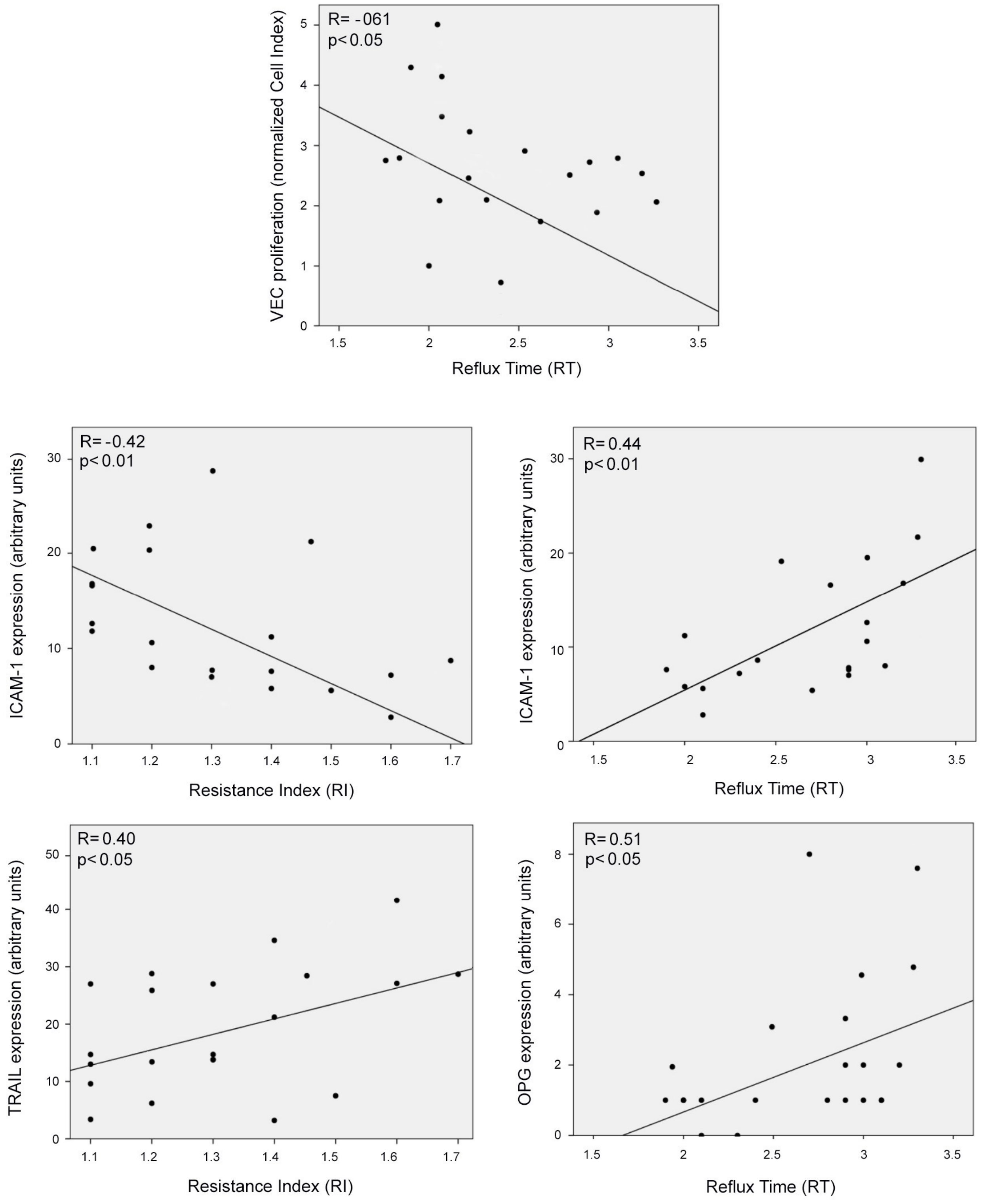

Supplement: Supplementary file 1 — The following main in vitro biological features of VEC: cell migration, proliferation index and baseline expression levels of ICAM-1, VCAM-1, OPG and TRAIL, were analyzed in correlation with the key hemodynamic parameters resistance index (RI) and reflux time (RT). The analysis of the correlations showed that ICAM-1 expression in VEC cultures was negatively correlated with RI (p=0.002) and positively correlated with RT (p=0.007) (Supplementary Figure 1). The spontaneous in vitro VEC proliferation was negatively correlated with RT (p=0.027), while the expression of OPG positively correlated with RT values (p=0.013). Moreover, TRAIL expression levels showed a significant (p=0.048) positive correlation with RI (Supplementary Figure 1). [file 561689.f1.pdf]
